# 862. Augmented Renal Clearance Is Associated with Favorable Discharge Outcomes in Burn ICU Patients

**DOI:** 10.1093/jbcr/irag033.332

**Published:** 2026-04-06

**Authors:** Talia R Arcieri, Jessica M Delamater, Christopher F O'Neil, Ana M Reyes, Avery M Hebert, Akshat Sanan, Shevonne S Satahoo, Joyce I Kaufman, Louis R Pizano, Carl I Schulman, Kenneth G Proctor

**Affiliations:** Miami Burn Center, Miami, FL; Miami Burn Center, Miami, FL; Miami Burn Center, Miami, FL; University of Miami, Miami, FL; Miami Burn Center, Miami, FL; Miami Burn Center, Miami, FL; Miami Burn Center, Miami, FL; Miami Burn Center, Miami, FL; Miami Burn Center, Miami, FL; Miami Burn Center, Miami, FL; Miami Burn Center, Miami, FL

## Abstract

**Introduction:**

Augmented renal clearance (ARC) may have implications for dosing of renally cleared medications. Limited data exist on its prevalence and impact in burn patients. We hypothesized that ARC is common in critically ill burn patients and associated with favorable outcomes.

**Methods:**

Patients admitted to a single burn intensive care unit (ICU) from July 2021-May 2025 who had 24-hour urine creatinine collection performed were retrospectively reviewed. 24-hour creatinine clearance (CrCl) was calculated, with ARC defined as CrCl ≥130 mL/min/1.73 m2. Clinical outcomes were compared between patients with and without ARC. Favorable discharge outcome was defined as discharge to home or inpatient rehabilitation.

**Results:**

Of 53 patients (67.9% male, mean age 43 years, mean total body surface area burned 30.1%), ARC was present in 31 (58.5%). Mean CrCl was 185 mL/min/1.73 m2 in patients with ARC and 94 mL/min/1.73 m2 in those without (*p*<.001). Patients with ARC were younger (*p*=.025). There were no significant differences between groups in early fluid or blood product administration, urine output or fluid balance during urine collection, in-hospital complications, or hospital or ICU length of stay (Table 1). Patients with ARC were more likely to have favorable discharge outcomes (*p*=.008) and had a trend towards decreased mortality (*p*=.188).

**Conclusions:**

ARC is common in burn ICU patients and associated with improved discharge outcomes. ARC may reflect a preserved physiologic reserve and compensatory renal response in critical illness. Though there were no differences in hospital complications or other outcomes, the population and event rates were both small. Prospective studies in larger populations are needed to better understand the etiology and impact of ARC in burn patients.

**Applicability of Research to Practice:**

ARC may impact medication dosing and outcomes.

**Funding for the study:**

N/A.

